# Association between intra- and peritumoral heterogeneity on B-mode ultrasound and overall survival in patients with liver metastases treated with immunotherapy

**DOI:** 10.1186/s41747-026-00771-6

**Published:** 2026-07-13

**Authors:** Littisha Lawrance, Ingrid Leguerney, Baya Benane-Benatsou, Jules Dupont, Samy Ammari, Alexandre Bône, Corinne Balleyguier, Aurélie Choucair, Christophe Massard, Nathalie Lassau, Amandine Crombé

**Affiliations:** 1 Biomaps, UMR1281, INSERM, Centre National de la Recherche Scientifique (CNRS), Commissariat à l’Energie Atomique (CEA), Université Paris Saclay, Villejuif, France; 2 Guerbet Research, Villepinte, France; 3 Department of Diagnostic Oncologic Imaging, Gustave Roussy Institute, Villejuif, France; 4 Département d’Innovation Thérapeutique et des Essais Précoce, Gustave Roussy, Villejuif, France; 5 Université Paris-Saclay, Villejuif, France; 6 Department of Cancer Medicine, Gustave Roussy, Villejuif, France; 7 Department of Radiology, Pellegrin University Hospital, Bordeaux, France; 8 Bordeaux Institute of Oncology, BRIC U1312, INSERM, Team ‘SARCOTARGET’, Bordeaux, France

**Keywords:** Immune checkpoint inhibitors, Immunotherapy, Liver neoplasms, Survival analysis, Ultrasonography

## Abstract

**Objective:**

Noninvasive prognostic biomarkers are needed for patients receiving immune checkpoint inhibitors (ICIs). We evaluated whether ultrasound-derived spatial heterogeneity indices (SHI) from intra- and peritumoral regions of liver metastases from solid cancers—measured at baseline, at first evaluation, and as longitudinal changes—are associated with overall survival (OS) in patients treated with ICIs.

**Materials and methods:**

This retrospectively included 27 consecutive patients (median age: 58 years) with liver metastases undergoing ICI therapy. B-mode ultrasound was performed at baseline (D0) and day 21. For each time point, voxel intensities within manually segmented intra- and peritumoral regions were clustered using k-means to compute a normalized SHI. Intratumoral, peritumoral, and combined (intra+peri) SHIs were extracted, and relative changes (ΔSHI) were calculated. Associations with overall survival (OS) were tested using univariable and multivariable Cox regression models.

**Results:**

At a median follow-up of 12.3 months, 92.6% patients had died. Baseline SHIs were not associated with OS. However, higher SHIs at D21 were associated with longer OS: intratumoral (hazard ratio (HR) = 0.39, *p* = 0.030), peritumoral (HR = 0.38, *p* = 0.029), and combined regions (HR = 0.28, *p* = 0.007). In contrast, larger decreases in intratumoral ΔSHI (≤ ‐3.9%) and combined ΔSHI (≤ ‐3.0%) were associated with worse OS (*p* = 0.0297 and 0.0339, respectively). In multivariable analysis, only the combined SHI at D21 > median remained independently predictive of longer OS (HR = 0.09, *p* = 0.021).

**Conclusion:**

The combined SHI at D21 was independently associated with OS in patients with liver metastases from ICI-treated solid cancers.

**Relevance statement:**

In this exploratory study, ultrasound-derived spatial heterogeneity indices estimated on intra- and peritumoral tissue on liver metastases offer a simple, contrast-free tool that was associated with overall survival in patients treated with immunotherapy, suggesting potential value for noninvasive risk stratification, pending validation in larger cohorts.

**Key Points:**

- The combined intratumoral and peritumoral ultrasound-based spatial heterogeneity index (SHI) at D21 was independently associated with OS, whereas dimensional metrics did not.
- Greater intratumoral, peritumoral, and combined SHI at D21, along with smaller decreases (or increases) in ΔSHI_combined_ and ΔSHI_intra_, were associated with longer OS.

**Graphical Abstract:**

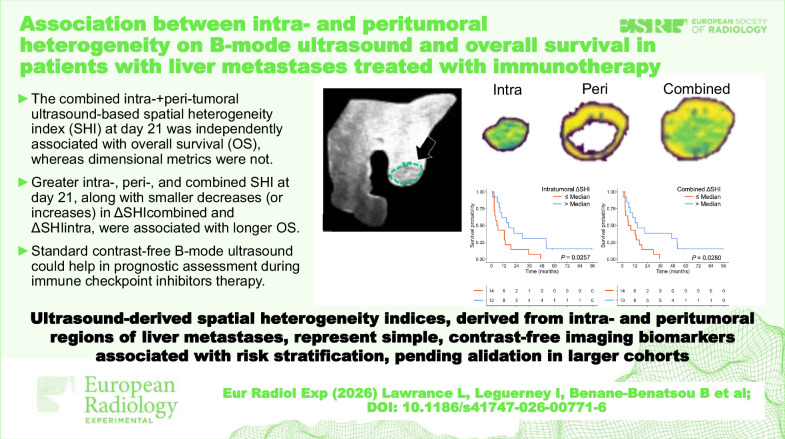

## Background

Immune checkpoint inhibitors (ICIs) have transformed the therapeutic landscape of advanced solid tumors, offering durable benefit to subsets of patients across cancer types [1]. However, objective response rates remain limited, ranging from < 20% in hepatocellular carcinoma and non-small cell lung cancer to about 50% in melanoma and Hodgkin lymphoma [2], highlighting the need for early, noninvasive biomarkers to identify responders. In addition to the heterogeneity of tumor cells within the tumor core, the structure and immune composition of the peritumoral microenvironment strongly influence therapeutic response [3, 4]. Distinct immune phenotypes, namely immune-inflamed, immune-excluded, and immune-desert, reflect differences in spatial immune organization and are predictive of benefit from ICI [3].

Indeed, accumulating evidence indicates that peritumoral regions harbor unique prognostic information, particularly through the presence and localization of tertiary lymphoid structures (TLS), which shape the local immune contexture [5–7]. The density and spatial positioning of TLS relative to the invasive margin have been associated with ICI efficacy across multiple cancer types [8–11]. Specifically, proximal intratumoral TLS are often linked to favorable outcomes, whereas distal peritumoral TLS in normal parenchyma correlate with poorer prognosis [12]. These findings underscore the biological importance of assessing both the tumor core and its periphery when evaluating the immune response to ICI.

Histopathologic and transcriptomic studies have characterized these immune and stromal architectures but remain invasive and prone to sampling bias [5]. Innovative quantitative imaging biomarkers offer a noninvasive alternative to capture global and spatially resolved tumor heterogeneity [13], potentially overcoming biases of purely dimensional assessments, which can be confounded by immune-related phenomena such as pseudoprogression or transient immune-cell infiltration during ICI therapy. Radiomics, which extracts large sets of quantitative features from medical images, has shown that both intra- and peritumoral texture features on computed tomography (CT), magnetic resonance imaging (MRI), or positron emission tomography (PET) can predict immune infiltration, programmed death-ligand 1 (PD-L1) expression, and survival outcomes under ICI [14–16]. However, conventional radiomics approaches rely on high-dimensional, black-box models, limiting their interpretability and reproducibility and motivating interest in more parsimonious, structurally interpretable heterogeneity descriptors [17]. One such clustering-based framework, previously described as a spatial organization metric derived from clustered parametric maps, provides a single quantitative measure of heterogeneity grounded in the spatial arrangement of image subregions [17]. In the present study, we adopt and adapt this previously reported methodological concept to ultrasound imaging.

Ultrasound, widely available, inexpensive, and free of ionizing radiation, is uniquely suited for repeated assessment of liver metastases. It can dynamically monitor tumor perfusion using dynamic contrast-enhanced (DCE) ultrasound, vascular remodeling, stiffness, and textural heterogeneity during treatment [18, 19]. Quantitative ultrasound parameters have already demonstrated correlations with immune-related vascular and microstructural changes, supporting their potential for early prediction of treatment response [20]. While CT and MRI radiomics have been extensively used to quantify intra- and peritumoral heterogeneity, such approaches in ultrasound remain limited, particularly in the delta-radiomics (longitudinal) setting.

The spatial heterogeneity index (SHI) was recently proposed as an interpretable, clustering-based metric quantifying image heterogeneity through the spatial organization and volumetric contribution of subregions within a lesion [17]. Unlike conventional radiomics models, which rely on hundreds to thousands of agnostic statistical descriptors (*i.e*., the radiomics features), the SHI provides a single, physically interpretable measure of tumor heterogeneity that captures the complexity of spatial organization rather than texture alone. While it has been validated in MRI perfusion maps and preclinical anti-angiogenic models, it has never been applied to ultrasound imaging or to immunotherapy monitoring.

Herein, we hypothesized that ultrasound-derived SHI values, computed on B-mode images of hepatic metastases, could serve as noninvasive markers of tumor and peritumoral heterogeneity reflective of immune-related tissue remodeling and effect since the first imaging revaluation. Therefore, our aim was to evaluate whether SHI from intra-, peritumoral, and combined regions of liver metastases from solid cancers—measured at baseline, at first evaluation, and as longitudinal changes—were associated with overall survival (OS) in patients treated with ICIs. This approach aims to bridge the gap between conventional radiomics and biologically meaningful, interpretable imaging features, leveraging the accessibility and dynamic potential of ultrasound for personalized immunotherapy response assessment.

## Methods

### Patients

This single-center retrospective observational exploratory study was approved by the Institutional Review Board of Gustave Roussy Institute (Villejuif, France) and performed in agreement with good clinical practice, applicable laws, and the Declaration of Helsinki. Written informed consent was waived by the Institutional Review Board due to its retrospective nature.

**Fig. 1:**
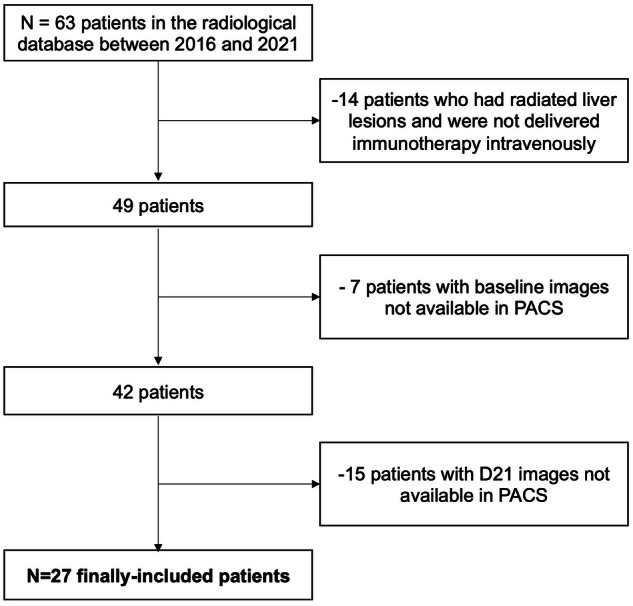
Study flowchart. D21, Day 21 after immunotherapy beginning; PACS, Picture Archiving and Communication System

Patients were identified starting from a radiological database of 63 adult patients with metastatic solid cancers who underwent regular liver ultrasonography to monitor response to systemic treatments between 2016 and 2021. All consecutive patients fulfilling the following inclusion criteria were included: (1) treated solely with intravenous ICI between 2016 and 2021 and followed-up at Gustave Roussy Institute; (2) with available B-mode image at baseline (D0) and at day 21 (D21) following treatment start available on our Picture Archiving and Communication System; and (3) at least one metastatic liver lesion (regardless of the primary cancer origin) entirely covered on ultrasonography at D0 and D21. It must be noted that patients were included in a prior study with different objectives, namely to monitor the response to treatment of various tumors using dynamic contrast-enhanced ultrasound [18]. Figure 1 shows the study flowchart.

Age, sex, primary cancer histological type, ICI treatment, and neutrophil-lymphocyte ratio (NLR, at D0 and D21) were extracted from medical records. The main study endpoint was the OS, which was defined as the time from the date of ICI beginning to death or last follow-up, censoring patients lost to follow-up at the end of data collection (September 2025). Secondary endpoint included progression-free survival (PFS), which was defined as the time from ICI initiation to disease progression (assessed according to RECIST v1.1 on scheduled protocol CT scans performed every 3 months) or last follow-up with patients lost to follow-up similarly censored at the end of data collection. OS was selected as the primary endpoint because it is objective, clinically meaningful, and less susceptible than PFS to assessment bias, imaging interval variability, and atypical response patterns such as pseudoprogression under immunotherapy. Moreover, in this cohort—largely enrolled in clinical trials with limited subsequent treatment lines—OS was considered the most robust reflection of global disease aggressiveness across heterogeneous tumor types, whereas PFS was analyzed as a secondary endpoint to provide complementary treatment-specific information.

### Ultrasound protocol

B-mode ultrasound examinations were performed by three senior radiologists (S.A., N.L., and C.B.) specialized in oncologic imaging using the Aplio i900 system (Canon Medical Systems) equipped with a PVI-475BX abdominal probe. The main acquisition parameters were: frame rate = 1; precision = 4; ApliPure = 1; dynamic range = 70 dB; and general frequency = 4 MHz.

Raw acquisitions were standardized to capture the entire metastasis at its largest section in the axial and sagittal planes. For each patient, a single target liver metastasis was selected for analysis, chosen as the best compromise between accessibility, image quality, lesion size, and representativeness. These images were exported in DICOM format to the SPYD annotation platform (Owkin, Paris, France). A senior radiologist with over 30 years of experience (N.L.) manually delineated this same hepatic target lesion at D0 and D21 on the axial image to define the intratumoral region of interest (ROI), blinded to the patient outcome. However, because the radiologist required knowledge of the baseline lesion location to identify and select the same lesion at D21, blinded assessment was not feasible. A peritumoral ROI was then automatically generated by applying a 5-mm dilation to the intratumoral ROI, followed by subtraction of the latter and manual refinement when necessary. Lastly, the intra- and peritumoral regions were merged to define a composite ROI encompassing both the tumor and its surrounding tissue.

For inter-patient comparability, ultrasound gray-level intensities were normalized according to the mean gray value of the subcutaneous fat, following a previously published method [21].

Furthermore, the tumor’s longest diameter (LD) was measured at D0 and D21.

### Heterogeneity quantification

To quantify tissue heterogeneity within ROIs on ultrasound, we applied a data-driven clustering approach followed by computation of the spatial heterogeneity index (SHI, formerly named H-index) [17, 22]. First, voxel intensities within each ROI were extracted and subjected to one-dimensional k-means clustering (with k = 3, established in prior works) [17, 22], grouping voxels with similar intensity values to delineate locally homogeneous subregions within the tissue. For each intensity cluster, spatially connected components were identified in the two-dimensional space, each corresponding to a contiguous group of voxels sharing similar intensity.

Three features were then computed for each cluster: (1) area fraction (*fA*), representing the proportion of pixels belonging to the cluster relative to the total ROI area; (2) N, the number of spatially disconnected components within the cluster; and (3) D, the mean Euclidean distance between the centroids of all components, describing the spatial dispersion of homogeneous regions.

The heterogeneity contribution of each cluster was defined as:

$${SHI}_{cluster}={fA}\times N\times D,$$

and the overall SHI for the ROI was calculated as the sum of all cluster contributions:

$${SHI}_{ROI}=\sum {SHI}_{cluster},$$

To account for inter-lesion variability in cluster number and spatial configuration, a normalized SHI was computed by weighting each cluster’s contribution by its relative *N*, *D*, and *fA* values across the ROI:

$${{SHI}}_{{normalized}}={\sum }_{i=1}^{k}\frac{{N}_{i}}{\mathop{\sum }_{j=1}^{k}{N}_{j}}\times \frac{{D}_{i}}{\mathop{\sum }_{j=1}^{k}{D}_{j}}\times {{fA}}_{i}$$

Because the SHI may scale with lesion volume, normalization by tumor area was also performed to minimize size-related bias [17, 22].

Overall, for each patient, the SHI (normalized for both gray-level intensities and lesion size) was measured in the intratumoral, peritumoral, and combined regions at D0 and D21. These measurements enabled calculation of the relative change in SHI between the two time points (named, ΔSHI_intra_, ΔSHI_peri_, and ΔSHI_combined_, respectively) expressed in percentage.

**Fig. 2:**
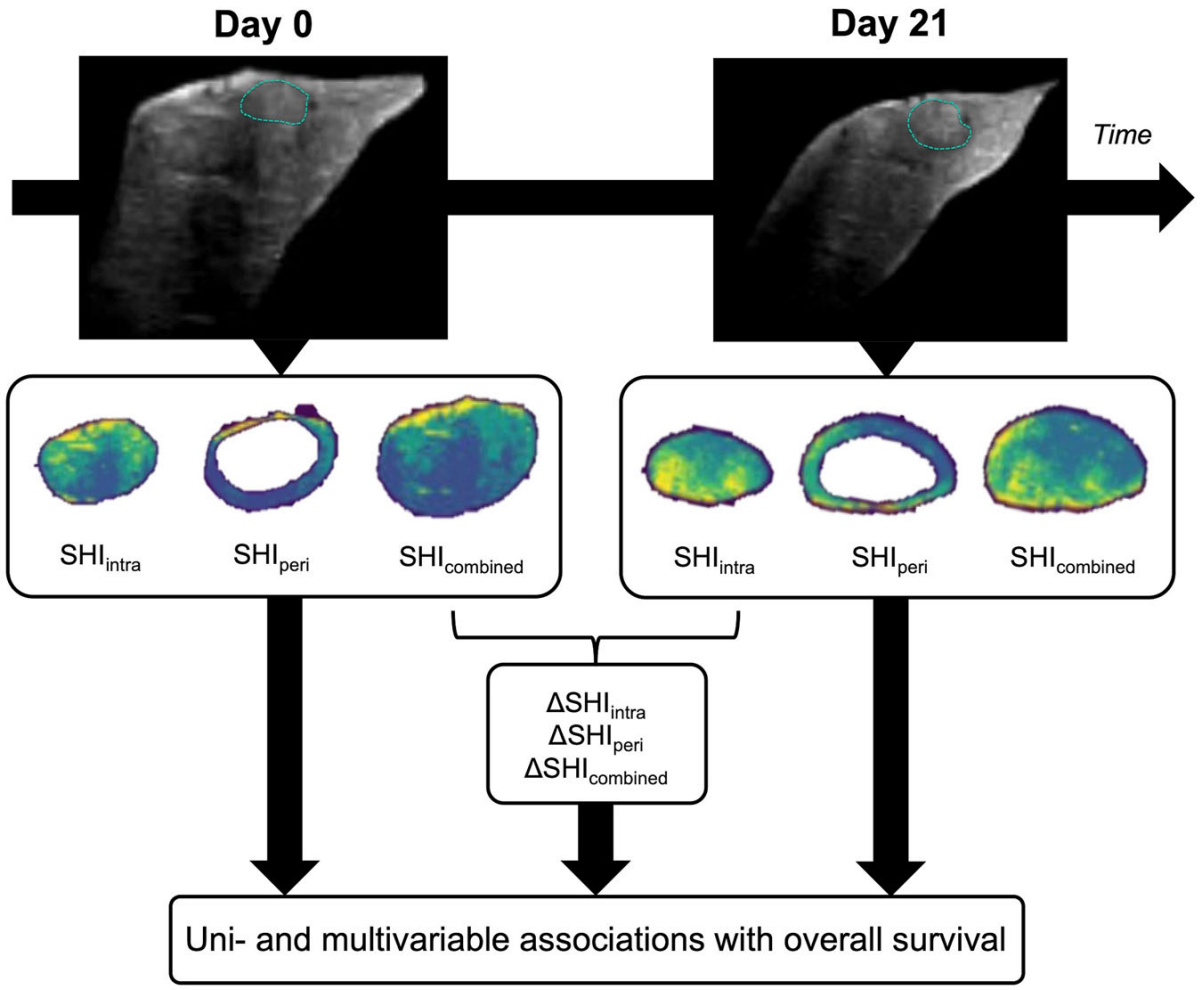
Workflow of raw image processing and heterogeneity analysis. Heterogeneity index (SHI) values were extracted at baseline (D0) and day 21 (D21) for intratumoral (intra), peritumoral (peri), and combined regions, followed by calculation of their relative changes

Figure 2 illustrates the image processing workflow and the subsequent statistical analysis.

### Statistical analysis

Statistical analyses were performed with R (v4.1.0). All tests were two-tailed; *p*-values < 0.05 defined statistical significance.

Categorical data were described as the number of patients and percentages. Numeric variables were described as median, interquartile range (IQR), and minimum-maximum range or mean ± standard deviation, as appropriate.

NLR, LD, intratumoral, peritumoral, and combined SHIs at D0 and D21 were compared using nonparametric paired Wilcoxon test (after verifying the lack of normality using the Shapiro–Wilk normality test). Correlations between these continuous measurements were assessed using the Spearman rank test. Associations with histological types were investigated with the Kruskal–Wallis test.

All continuous measurements at each time point, along with their relative changes, were subsequently dichotomized using the cohort-wide median to facilitate clinical interpretation (*i.e*., low *versus* high) while avoiding overfitting through optimized cut-off selection. OS was then analyzed using Kaplan–Meier estimates and compared between groups using the log-rank test. Univariable Cox proportional hazards models were used to estimate hazard ratios (HRs) and 95% CIs. Lastly, a multivariable Cox regression model was performed including all measurements with a univariable *p*-value < 0.05 and stratified by age, sex, and histological type. Regarding PFS, we performed analogous univariable analyses using Kaplan–Meier survival estimates and compared groups with the log-rank test. In parallel, univariable survival analyses for OS and PFS were also performed using these variables as continuous measures in Cox proportional hazards models. Correction for multiple testing was performed using the Benjamini–Hochberg procedure. Patients with any missing data for variables included in the multivariable analysis were excluded.

## Results

### Patient characteristics

Overall, 27 patients were finally included (14/27 [51.9%] women, median age: 58 years, IQR: 44–66, range: 29–81) (Table 1). Figure 1 shows the study flowchart. The most frequent histological type was colorectal cancer (7/27, 25.9%), followed by melanoma (6/27, 22.2%). The most frequent ICI regimen was pembrolizumab (12/27, 44.4%). The average size of the liver lesions at baseline was 42.4 ± 27 mm. Regarding survival, 25/27 (92.6%) patients demonstrated progression, and 25/27 (92.6%) patients died during the follow-up. The median OS time was 12.3 months, 95% CI: 6.2–21.4. No patient died or was lost to follow-up during the first 21 days after initiation of ICI therapy.

**Table 1:** Patient characteristics

Characteristics	Patients (*n* = 27)
Sex	
Men	13/27 (48.1)
Women	14/27 (51.9)
Age (years)	58 [44–66] (29–81)
Histological type of primary cancer	
Angiosarcoma	1/27 (3.7)
Breast cancer	1/27 (3.7)
Hepatocellular carcinoma	4/27 (14.8)
Colorectal cancer	7/27 (25.9)
Dedifferentiated liposarcoma	1/27 (3.7)
Kidney cancer	4/27 (14.8)
Leiomyosarcoma	2/27 (7.4)
Melanoma	6/27 (22.2)
Malignant solitary	1/27 (3.7)
ICI type	
Dual-checkpoint blockade	6/27 (22.2)
Anti–PD-1/PD-L1 monotherapy	21/27 (77.8)
LD at D0 (mm)	33 [25–54.5] (10–125)
NLR at D0	3.54 [2.46–4.13] (0.15–7.20)
Intratumoral SHI at D0	0.109 [0.102–0.112] (0.047–0.121)
Peritumoral SHI at D0	0.112 [0.108–0.117] (0.071–0.129)
Combined SHI at D0	0.107 [0.105–0.112] (0.1–0.121)
LD at D21 (mm)	37 [26–56.5] (14–123)
NLR at D21	3.53 [2.42–6.09] (1.27–34.81)
Intratumoral SHI at D21	0.103 [0.098–0.108] (0.065–0.122)
Peritumoral SHI at D21	0.113 [0.105–0.117] (0.097–0.121)
Combined SHI at D21	0.107 [0.101–0.112] (0.083–0.123)
ΔLD (%)	+7.5% [-2.1 + 27.9] (-26.7 + 67.9)
ΔNLR (%)	+6.9% [-17.3 + 59.7] (-54.1 + 1,085.2)
Intratumoral ΔSHI (%)	+0.56% [-7.78 + 6.89] (-17.28 + 66.3)
Peritumoral ΔSHI (%)	-3.92% [-7.43 + 3.14] (-34.79 + 36.54)
Combined ΔSHI (%)	-2.96% [-7.86 + 3.47] (-22.20 + 15.01)

Data are number of patients with percentages in parentheses for categorical variables. Data are median, interquartile range (in brackets) and minimum-maximum range (in parentheses) for numeric variables

*Δ* Relative change, *ICI* Immune checkpoint inhibitor, *LD* Longest diameter, *NLR* Neutrophil-lymphocyte ratio, *SHI* Spatial heterogeneity index

### Understanding SHIs

**Fig. 3:**
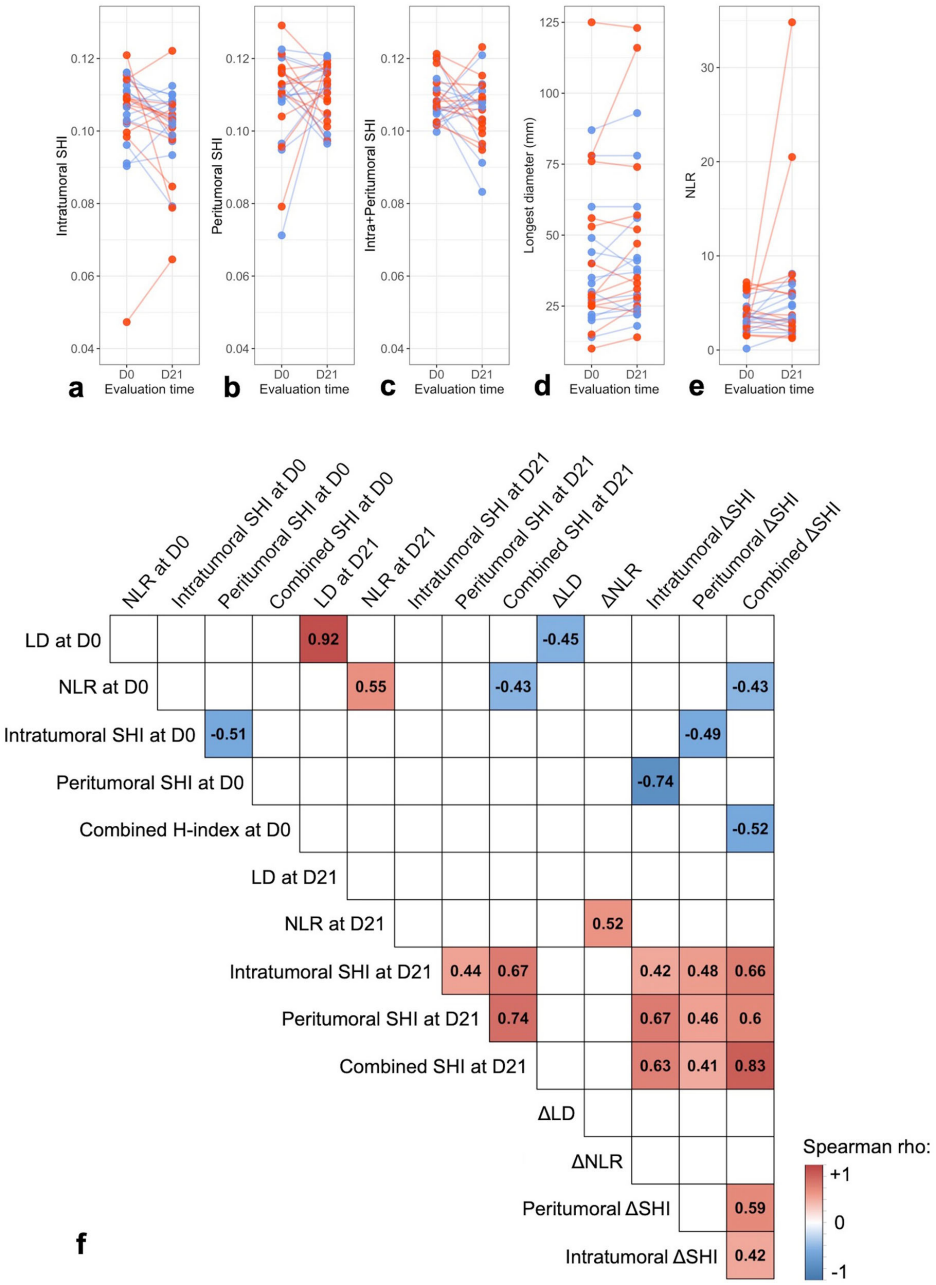
Exploratory analysis of heterogeneity indices (SHIs) from liver metastases, their temporal changes, and their association with the neutrophil-to-lymphocyte ratio (NLR) and tumor longest diameter (LD). Panels show changes in (**a**) intratumoral SHI, (**b**) peritumoral SHI, (**c**) combined intra- and peritumoral SHI, (**d**) LD, and (**e**) NLR between baseline (day 0, D0) and day 21 (D21) after initiation of immunotherapy. Each patient’s trajectory is represented by a thin line. Color coding distinguishes patients who died within 1 year (red) from those alive at 1 year (blue). **f** Correlation matrix showing significant associations between the different parameters and their relative changes, based on Spearman’s rank correlation. Only significant correlations are displayed, with the numeric values indicating Spearman’s rho within each cell

On average, the peritumoral SHI slightly increased during treatment (relative change: +0.56% IQR: -7.78% +6.89%, range: -17.28% +66.30%) while the combined and intratumoral SHIs slightly decreased (relative change: -3.92%, IQR: -7.43% +3.14%, range: -34.79% +36.54%; and -2.96% IQR: -7.86% +3.47%, range: -22.20% +15.01%, respectively). No associations were observed between the intratumoral, peritumoral, and combined SHIs at D0 and the histological types (*p*-value range: 0.135–0.332, Kruskal–Wallis test). Figure 3a–e illustrates the changes in the measurements between D0 and D21. No significant changes were observed (*p*-value range: 0.055 (for LD) to 0.8779 (for peritumoral SHI)). Figure 3f shows the significant correlations between the different measurements and their changes. Notably, the size at D0, D21, and its change were never correlated with the SHIs. The NLR at baseline was negatively correlated with the combined SHI at D21 (ρ = -0.43, *p* = 0.025) and the ΔSHI_combined_ (ρ = -0.43, *p* = 0.026). Regarding the baseline measurements, we observed a negative correlation between the intratumoral and the peritumoral SHIs (ρ = -0.51, *p* = 0.007). Regarding the D21 measurements, strong positive correlations were found between all SHIs (*p*-value range: < 0.001–0.021 with rho between 0.44 (intratumoral *versus* peritumoral SHIs) and 0.74 (combined *versus* peritumoral)). Regarding the changes in these measurements, significant correlations were observed between ΔSHI_combined_ and ΔSHI_peri_ (ρ = 0.59, *p* = 0.002) and ΔSHI_intra_ (ρ = 0.42, *p* = 0.032). Furthermore, strong positive correlations were found between all SHIs at D21 and their relative changes (*p*-value range: < 0.001–0.035, with ρ between 0.41 (combined SHI at D21 and ΔSHI_intra_) and 0.83 (combined SHI at D21 and ΔSHI_combined_)).

### Associations with survivals

Table 2 presents the univariable survival analysis. Neither the patient characteristics nor any of the baseline measurements were associated with OS. However, the intratumoral, peritumoral and combined SHIs measured at D21 were significantly associated with OS, with higher values (> median value, *i.e*., 0.103, 0.113 and 0.107, respectively) leading to lower risk of death (HR = 0.39, 95% CI: 0.16–0.91, *p* = 0.030; HR = 0.38, 95% CI: 0.16–0.91, *p* = 0.029; and HR = 0.28, 95% CI: 0.11–0.71, *p* = 0.007, respectively).

**Table 2:** Univariable overall survival (OS) analysis

Characteristics	No. at risk	No. of events	Survival probability at 1 year (95% CI)	Median OS (months, 95% CI)	Log-rank *p*-value	Adjusted Log-rank *p*-value	HR (95% CI)	*p*-value
Age								
≤ Median (58 years)	15	13	60 (39.69–90.7)	14.4 (7.4–52)	0.208	0.360	Ref.	-
> Median	12	12	41.67 (21.33–81.38)	6.8 (2.7–.)			1.66 (0.75–3.65)	0.209
Sex								
Men	13	12	53.85 (32.55–89.07)	12.3 (5.3–NA)	0.879	0.879	Ref.	-
Women	14	13	50 (29.61–84.42)	11.1 (5.6–.)			1.06 (0.48–2.33)	0.887
Histological type								
Other	6	6	16.67 (2.78–99.74)	4.9 (2.7–.)	0.056	0.159	Ref.	-
Hepatocellular carcinoma	4	4	25 (4.58–100)	4 (2.5–.)			0.72 (0.19–2.68)	0.627
Colorectal cancer	7	6	57.14 (30.08–100)	12.3 (6.2–.)			0.33 (0.1–1.08)	0.067
Kidney cancer	4	4	75 (42.59–100)				0.35 (0.09–1.31)	0.119
Melanoma	6	5	83.33 (58.27–100)	38.1 (12.3–.)			0.18 (0.05–0.66)	0.009*
ICI type								
Dual-checkpoint blockade	6	5	83.33 (58.27–100)	38.1 (12.3–.)	0.058	0.159	Ref.	-
Anti–PD-1/PD-L1 monotherapy	21	20	42.86 (26.15–70.23)	7.4 (4.2–18.6)			2.55 (0.93–6.95)	0.068
NLR at D0								
≤ Median (3.5)	14	12	64.29 (43.51–94.99)	15.4 (8.2–.)	0.092	0.194	Ref.	-
> Median	13	13	38.46 (19.34–76.5)	6.2 (2.8–.)			1.97 (0.89–4.39)	0.096
LD at D0								
≤ Median (33 mm)	14	13	50 (29.61–84.42)	10.2 (6.2–52.4)	0.556	0.661	Ref.	-
> Median	13	12	53.85 (32.55–89.07)	12.3 (5.3–.)			1.27 (0.57–2.82)	0.558
Intratumoral SHI at D0								
≤ Median (0.109)	14	13	50 (29.61–84.42)	10.2 (6.2–52)	0.860	0.879	Ref.	-
> Median	13	12	53.85 (32.55–89.07)	12.3 (4.2–.)			0.93 (0.42–2.06)	0.861
Peritumoral SHI at D0								
≤ Median (0.112)	14	13	64.29 (43.51–94.99)	16.5 (9.9–52.4)	0.349	0.549	Ref.	-
> Median	13	12	38.46 (19.34–76.5)	7.4 (5.3–.)			1.46 (0.66–3.23)	0.356
Combined SHI at D0								
≤ Median (0.107)	14	13	64.29 (43.51–94.99)	13.4 (6.2–52)	0.652	0.729	Ref.	-
> Median	13	12	38.46 (19.34–76.5)	8.2 (2.8–.)			1.2 (0.54–2.64)	0.656
NLR at D21								
≤ Median (3.5)	14	12	57.14 (36.3–89.94)	12.8 (7.4–.)	0.120	0.228	Ref.	-
> Median	13	13	46.15 (25.66–83.02)	9.9 (2.8–.)			1.9 (0.84–4.32)	0.125
LD at D21								
≤ Median (37 mm)	14	13	50 (29.61–84.42)	10.2 (6.2–52.4)	0.505	0.640	Ref.	-
> Median	13	12	53.85 (32.55–89.07)	12.3 (2.7–.)			1.31 (0.59–2.91)	0.502
Intratumoral SHI at D21								
≤ Median (0.103)	14	14	42.86 (23.41–78.47)	5.9 (3.3–21.4)	0.025*	0.106	Ref.	-
> Median	13	11	61.54 (40.04–94.58)	14.4 (8.2–.)			0.39 (0.16–0.91)	0.030*
Peritumoral SHI at D21								
≤ Median (0.113)	14	14	42.86 (23.41–78.47)	5.4 (3.3–29.5)	0.024*	0.106	Ref.	-
> Median	13	11	61.54 (40.04–94.58)	16.4 (8.2–.)			0.38 (0.16–0.91)	0.029*
Combined SHI at D21								
≤ Median (0.107)	14	14	42.86 (23.41–78.47)	5.4 (3.3–18.6)	0.005**	0.089	Ref.	-
> Median	13	11	61.54 (40.04–94.58)	21.4 (8.2–.)			0.28 (0.11–0.71)	0.007*
ΔNLR								
≤ Median (+6.9%)	14	14	28.57 (12.48–65.41)	5.9 (4.2–46.8)	0.082	0.194	Ref.	-
> Median	13	11	76.92 (57.11–100)	16.4 (12.3–.)			0.5 (0.22–1.11)	0.089
ΔLD								
≤ Median (+7.5%)	14	14	57.14 (36.3–89.94)	12.8 (5.6–35.4)	0.376	0.549	Ref.	-
> Median	13	11	46.15 (25.66–83.02)	8.2 (4.2–.)			0.69 (0.3–1.58)	0.382
Intratumoral ΔSHI								
> Median	13	11	61.54 (40.04–94.58)	16.4 (8.2–.)	0.026*	0.106	Ref.	-
≤ Median (-3.9%)	14	14	42.86 (23.41–78.47)	5.8 (3.3–35.4)			2.57 (1.1–6.04)	0.030*
Peritumoral ΔSHI								
> Median	13	12	53.85 (32.55–89.07)	12.3 (6.2–.)	0.456	0.618	Ref.	-
≤ Median (+0.6%)	14	13	50 (29.61–84.42)	9.9 (5.3–52)			1.35 (0.61–2.99)	0.460
Combined ΔSHI								
> Median	13	11	61.54 (40.04–94.58)	14.4 (7.4–.)	0.028*	0.160	Ref.	-
≤ Median (-3.0%)	14	14	42.86 (23.41–78.47)	6.9 (3.3–29.5)			2.61 (1.08–6.32)	0.034*

*Δ* Relative change, *CI* Confidence interval, *HR* Hazard ratio, *ICI* Immune checkpoint inhibitor, *LD* Longest diameter, *NLR* Neutrophil-lymphocyte ratio, *Ref*. Reference level, *SHI* Spatial heterogeneity index

* *p* < 0.05, ** *p* < 0.005, *** *p* < 0.001. Significant results are in bold. When the upper bound of the 95% CI of the median survival time is not reached, the value is replaced by a dot. Because of small effectives, the various sarcoma histotypes and breast cancers were merged in an ‘other’ group

**Fig. 4:**
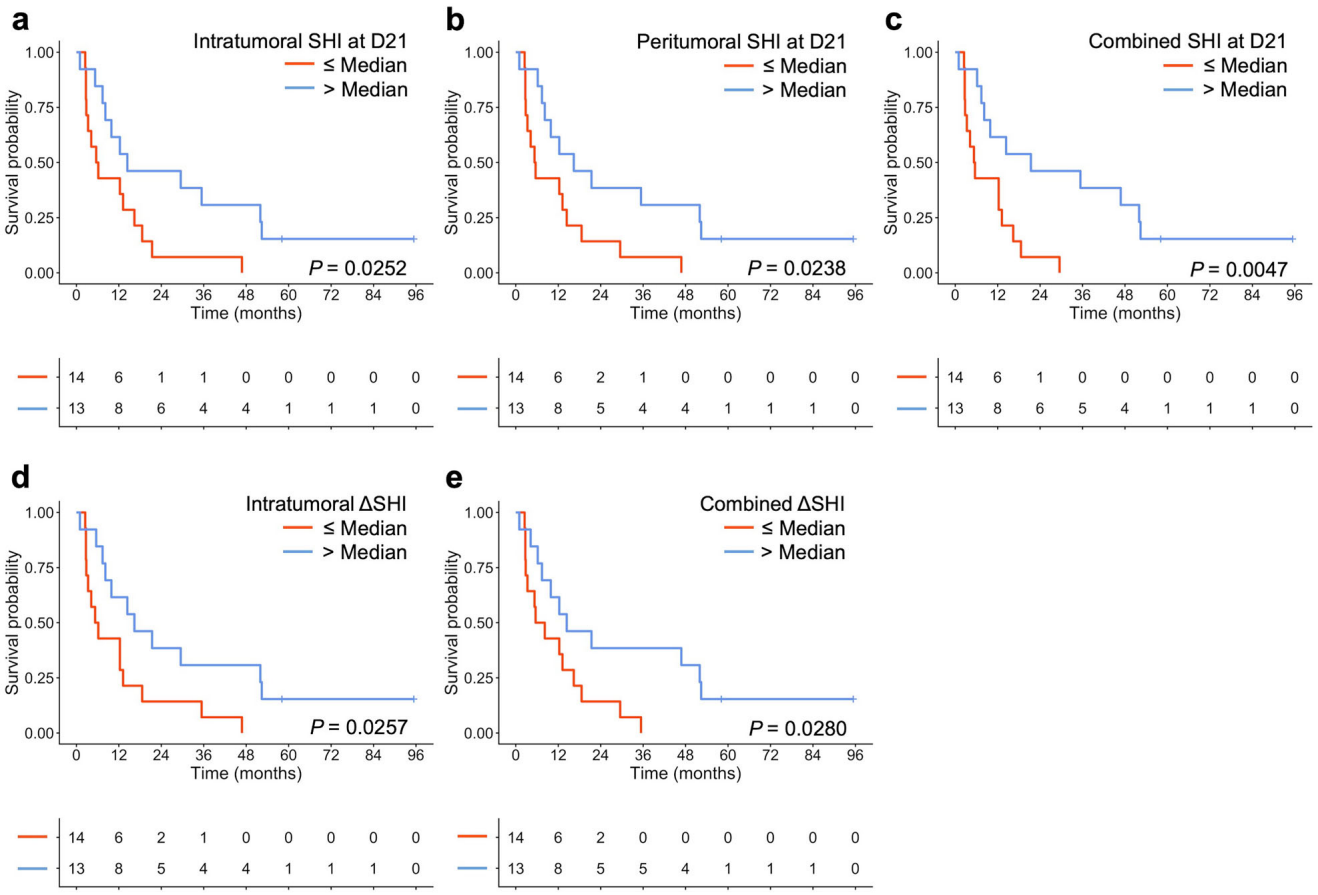
Kaplan–Meier curves for overall survival (OS) for 5 prognostic measurements identified in the univariable analysis, namely (**a**) intratumoral heterogeneity index (SHI) at day 21 (D21), (**b**) peritumoral SHI at D21, (**c**) combined SHI at D21, (**d**) relative change (Δ) in intratumoral SHI, and (**e**) relative change (Δ) in combined SHI; *p*-values correspond to the Log-rank test

Furthermore, while the LD and NLR at D0 and D21, as well as their relative changes, were not linked to patient survival, ΔSHI_intra_ and ΔSHI_combined_ were significantly associated with OS, with larger decrease (*i.e*., ≤ -3.9% and ≤ -3%, respectively) being linked to worse OS (HR = 2.57, 95% CI: 1.10–6.04, *p* = 0.030; and HR = 2.61, 95% CI: 1.08–6.32, *p* = 0.034, respectively). However, none of these associations remained significant after adjusting for multiple comparisons. Figure 4 shows the Kaplan–Meier curves of these 5 significant metrics. When analyzed as continuous variables, no SHI-derived metrics were significantly associated with OS after correction for multiple testing, although nominal associations were observed for intratumoral SHI at D21 and ΔSHI_combined (both *p* = 0.022).

**Fig. 5:**
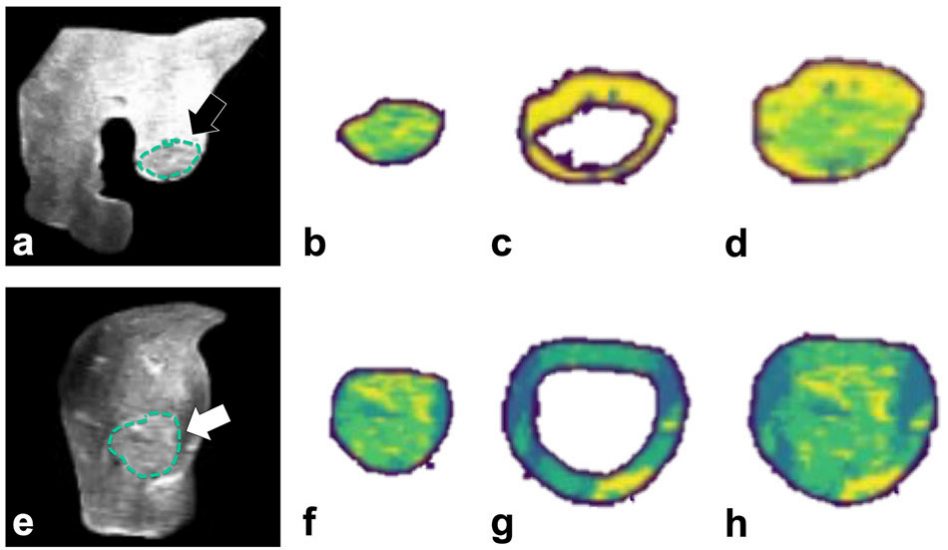
Representative cases illustrating favorable and unfavorable outcomes under immunotherapy. Top row (favorable outcome): 77-year-old man with metastatic melanoma treated with dual-agent immunotherapy. **a** Axial B-mode ultrasound at day 21 showing the segmented target lesion (dashed green line, black arrow). **b** Intratumoral segmentation, (**c**) peritumoral rim, and (**d**) combined region. At day 21, all SHIs were high (above the median). The patient achieved a durable response lasting 19 months and died 52.4 months later. Bottom row (unfavorable outcome): 65-year-old man treated with single-agent immunotherapy. **e** Axial B-mode ultrasound at day 21 showing the segmented target lesion (dashed green line, white arrow). **f** Intratumoral segmentation, (**g**) peritumoral rim, and (**h**) combined region. At day 21, all SHIs were low (below the median). The patient experienced disease progression after 5 months and died 13 months later

A multivariable analysis was finally conducted including these 5 measurements identified in the univariable analysis and adjusted for age, sex, histological type, and ICI treatment (Table 3). The combined SHI at D21 remained independently associated with OS (multivariable HR = 0.09, 95% CI: 0.01–0.70, *p* = 0.021 for SHI at D21 > median [0.107] compared to ≤ median). The other significant variable was anti-PD1/PD-L1 monotherapy (HR = 10.45, 95% CI: 1.37–79.72, *p* = 0.024, compared to dual-checkpoint blockage). Figure 5 illustrates these findings with two patients with opposite outcome.

**Table 3:** Summary of the multivariable survival analysis

Characteristics	Multivariable HR (95% CI)	*p*-value
Age		
≤ Median (58 years)	Ref.	–
> Median	3.58 (0.58–22.14)	0.170
Sex		
Men	Ref.	–
Women	0.84 (0.17–4.06)	0.825
Histological type		
Other	Ref.	–
Hepatocellular carcinoma	1.61 (0.20–13.03)	0.654
Colorectal cancer	0.35 (0.08–1.49)	0.156
Kidney cancer	0.24 (0.05–1.23)	0.087
Melanoma	NA (NA–NA)^§^	–
ICI type		
Dual-checkpoint blockade	Ref.	–
Anti–PD-1/PD-L1 monotherapy	10.45 (1.37–79.72)	0.024*
Intratumoral SHI at D21		
≤ Median (0.103)	Ref.	–
> Median	0.57 (0.14–2.34)	0.438
Peritumoral SHI at D21		
≤ Median (0.113)	Ref.	–
> Median	1.03 (0.17–6.25)	0.973
Combined SHI at D21		
≤ Median (0.107)	Ref.	–
> Median	0.09 (0.01–0.70)	0.021*
Intratumoral ΔSHI		
> Median	Ref.	–
≤ Median (-3.9%)	0.46 (0.09–2.36)	0.355
Combined ΔSHI		
> Median	Ref.	–
≤ Median (-3.0%)	0.21 (0.04–1.17)	0.075

*Δ* Relative change, *CI* Confidence interval, *HR* Hazard ratio, *ICI* Immune checkpoint inhibitor, *Ref*. Reference level, *SHI* Spatial heterogeneity index

* *p* < 0.05. Significant results are in bold

^§^ The Cox regression could not converge to estimate the HR for the melanoma histotype with the other group as reference

Table 4 summarizes the univariable analysis for PFS. No significant associations were observed between PFS and any SHI- or LD-derived metrics, regardless of time point or measurement location. The only significant associations were with histological type (*p* = 0.047; melanoma associated with longer PFS, HR = 0.15, 95% CI: 0.03–0.67, *p* = 0.013), ICI regimen (longer PFS with dual-checkpoint blockade, HR = 4.04, 95% CI: 1.13–14.41, *p* = 0.032), and NLR at D21 (shorter PFS when NLR exceeded the median value of 3.5, HR = 2.25, 95% CI: 1.00–5.05, *p* = 0.049). However, none of these associations remained significant after adjusting for multiple comparisons. Similarly, no imaging-derived variables were significantly associated with PFS after correction, while a nominal association was observed for NLR at D21 (*p* = 0.024).

**Table 4:** Univariable progression-free survival (PFS) analysis

Characteristics	No. at risk	No. of events	Survival probability at 1 year (95% CI)	Median PFS (months, 95% CI)	Log-rank *p*-value	Adjusted Log-rank *p*-value	HR (95% CI)	*p*-value
Age								
≤ Median (58 years)	15	13	10 (1.8–55.63)	2.6 (1.3–.)	0.621	0.753	Ref.	-
> Median	12	12	0 (.–.)	2.4 (1.4–.)			1.22 (0.55–2.73)	0.629
Sex								
Men	13	12	7.69 (1.17–50.57)	2.6 (2.1–.)	0.162	0.545	Ref.	-
Women	14	13	7.14 (1.08–47.22)	1.8 (1.3–4.1)			1.82 (0.78–4.22)	0.167
Histological type								
Other	6	6	0 (.–.)	1.7 (1.2–.)	0.047*	0.296	Ref.	-
Hepatocellular carcinoma	4	4	0 (.–.)	2.4 (1.3–.)			0.61 (0.17–2.21)	0.451
Colorectal cancer	7	6	14.29 (2.33–87.69)	2.7 (2.1–.)			0.38 (0.12–1.23)	0.108
Kidney cancer	4	4	0 (.–.)	0.8 (0.2–.)			0.94 (0.24–3.63)	0.931
Melanoma	6	5	16.67 (2.78–99.74)	12.3 (1.3–.)			0.15 (0.03–0.67)	0.013*
ICI type								
Dual-checkpoint blockade	6	5	16.67 (2.78–99.74)	12.3 (1.3–NA)	0.021*	0.296	Ref.	-
Anti–PD-1/PD-L1 monotherapy	21	20	4.76 (0.7–32.25)	2.3 (1.3–2.9)			4.04 (1.13–14.41)	0.032*
NLR at D0								
≤ Median (3.5)	14	12	14.29 (3.96–51.54)	2.4 (1.4–.)	0.673	0.753	Ref.	-
> Median	13	13	0 (.–.)	2.5 (1.3–.)			1.2 (0.54–2.64)	0.655
LD at D0								
≤ Median (33 mm)	14	13	0 (.–.)	2.2 (1.3–.)	0.943	0.943	Ref.	-
> Median	13	12	7.69 (1.17–50.57)	2.6 (1.3–.)			0.98 (0.44–2.17)	0.956
Intratumoral SHI at D0								
≤ Median (0.109)	14	13	0 (.–.)	2.5 (1.4–.)	0.635	0.753	Ref.	-
> Median	13	12	7.69 (1.17–50.57)	2.5 (1.3–.)			0.83 (0.37–1.83)	0.637
Peritumoral SHI at D0								
≤ Median (0.112)	14	13	0 (.–.)	2.8 (2.2–.)	0.289	0.640	Ref.	-
> Median	13	12	7.69 (1.17–50.57)	2.1 (1.2–.)			1.54 (0.69–3.43)	0.290
Combined SHI at D0								
≤ Median (0.107)	14	13	7.14 (1.08–47.22)	2.7 (2.2–19.3)	0.387	0.669	Ref.	-
> Median	13	12	0 (.–.)	2.1 (1.2–.)			1.42 (0.63–3.17)	0.397
NLR at D21								
≤ Median (3.5)	14	12	10.71 (1.94–59.29)	3.4 (2.2–.)	0.045*	0.296	Ref.	-
> Median	13	13	0 (.–.)	1.3 (1.1–.)			2.25 (1–5.05)	0.049*
LD at D21								
≤ Median (37 mm)	14	13	0 (.–.)	2.5 (2.1–.)	0.757	0.799	Ref.	-
> Median	13	12	7.69 (1.17–50.57)	2.3 (1.3–.)			1.15 (0.52–2.54)	0.734
Intratumoral SHI at D21								
≤ Median (0.103)	14	14	0 (.–.)	2.5 (1.4–4.6)	0.172	0.545	Ref.	-
> Median	13	11	10.26 (1.7–61.8)	2.5 (1.3–.)			0.56 (0.24–1.31)	0.182
Peritumoral SHI at D21								
≤ Median (0.113)	14	14	0 (.–.)	2.5 (2.2–5.6)	0.662	0.753	Ref.	-
> Median	13	11	11.54 (2.1–63.45)	2.1 (1.2–.)			0.84 (0.38–1.87)	0.667
Combined SHI at D21								
≤ Median (0.107)	14	14	0 (.–.)	2.2 (1.3–4.6)	0.144	0.545	Ref.	-
> Median	13	11	11.54 (2.1–63.45)	2.9 (1.3–.)			0.55 (0.24–1.23)	0.145
ΔNLR								
≤ Median (+6.9%)	14	14	0 (.–.)	2.4 (2.1–5.6)	0.518	0.753	Ref.	-
> Median	13	11	11.54 (2.1–63.45)	2.6 (1.1–.)			0.77 (0.34–1.74)	0.528
ΔLD								
≤ Median (+7.5%)	14	14	0 (.–.)	2.5 (1.4–4.6)	0.558	0.753	Ref.	-
> Median	13	11	11.54 (2.1–63.45)	2.2 (1.2–.)			0.79 (0.35–1.75)	0.556
Intratumoral ΔSHI								
> Median	14	14	0 (.–.)	2.4 (1.4–4.6)	0.205	0.556	Ref.	-
≤ Median (-3.9%)	13	11	10.26 (1.7–61.8)	2.6 (1.2–.)			0.58 (0.25–1.37)	0.213
Peritumoral ΔSHI								
> Median	14	13	7.14 (1.08–47.22)	2.4 (1.3–4.6)	0.363	0.669	Ref.	-
≤ Median (+0.6%)	13	12	0 (.–.)	2.7 (1.3–.)			0.69 (0.31–1.54)	0.366
Combined ΔSHI								
> Median	14	14	0 (.–.)	2.4 (1.4–4.6)	0.303	0.640	Ref.	-
≤ Median (-3.0%)	13	11	11.54 (2.1–63.45)	2.7 (1.3–.)			0.66 (0.29–1.47)	0.309

*Δ* Relative change, *CI* Confidence interval, *HR* Hazard ratio, *ICI* Immune checkpoint inhibitor, *LD* Longest diameter, *NLR* Neutrophil-lymphocyte ratio, *Ref*. Reference level, *SHI* Spatial heterogeneity index

* *p* < 0.05, ** *p* < 0.005, *** *p* < 0.001. Significant results are in bold. When the bounds of the 95% CI of the median survival time and 1-year survival probability are not reached, the value is replaced by a dot. Because of small effectives, the various sarcoma histotypes and breast cancers were merged in an ‘other’ group

## Discussion

This exploratory study investigated whether tumor heterogeneity derived from non-contrast B-mode ultrasound could provide prognostic information in patients with liver metastases undergoing ICI. Specifically, it assessed whether the spatial organization complexity quantified by the SHI within the intratumoral and peritumoral regions was linked to OS at D21 (*i.e*., early, first imaging revaluation). Using a retrospective, multicancer cohort of 27 patients, the study demonstrated that a higher combined SHI value measured at D21 was independently associated with improved OS.

In this study, heterogeneity quantification from standard B-mode ultrasound revealed that peritumoral, intratumoral, and combined SHI values measured at D21 were significantly associated with OS, while no baseline association was observed. Patients with higher intratumoral, peritumoral, and combined heterogeneity (above the cohort median) at D21 showed higher OS than those below the cohort median. Furthermore, in multivariable analysis, the combined intra+peritumoral SHI at D21 remained the sole ultrasound-based independent feature linked to OS, suggesting a potential prognostic interest of integrating peritumoral heterogeneity in addition to intratumoral heterogeneity. Because ΔSHI is mathematically dependent on baseline and D21 values, its interpretation as an independent biomarker is limited, and the lack of significance in multivariable analysis suggests that SHI at D21 may capture the primary prognostic signal.

ΔSHI_combined_ ΔSHI_intra_ shrinkages greater than their median (-3% and -3.9%, respectively) between D0 and D21 were associated with worse survival. These findings support that structural reorganization captured in the intra- and peritumoral compartment at D21 reflects the biological activity of the immune response under checkpoint blockade. This temporal pattern is consistent with prior DCE ultrasonography data showing that a ≥ 45% reduction in perfusion area under the curve at D21 correlated with improved OS in ICI-treated patients [18], and with histologic evidence of immune-mediated vascular remodeling within the same time frame [20].

Interestingly, while significant associations were found between OS and SHIs, neither LD and NLR at baseline, D21 nor their relative changes were correlated with this patient outcome.

Our results reinforce the complementary prognostic role of spatial heterogeneity at the tumor margin, echoing pathological studies that link mature peritumoral TLS with prolonged recurrence-free survival in hepatocellular carcinoma [10] and perihilar cholangiocarcinoma [9]. In pathological studies involving colorectal cancer liver metastases, the immune class combining intratumoral and peritumoral TLS scores outperformed clinical risk systems for outcome prediction [8], highlighting the importance of assessing both compartments. However, the lack of direct histopathologic or molecular correlation limits the biological interpretation of SHI, which should be considered as an imaging surrogate of spatial heterogeneity rather than a direct measure of tumor or immune composition. Comparable imaging findings were recently reported on CT and MRI, where radiomic models integrating intra- and peritumoral features achieved higher predictive accuracy for ICI response (area under the curve = 0.82–0.86) than those restricted to the tumor core [15, 23, 24]. PET-CT studies combining tumor and peritumoral radiomic signatures similarly achieved a validation area under the curve = 0.819 and were correlated with CD8⁺ T-cell infiltration [15]. The absence of a baseline association in our study further supports the concept that dynamic, therapy-induced heterogeneity changes (and not pre-existing texture) carry the strongest prognostic signal, consistent with delta-radiomics observations in CT and MRI under immunotherapy [14, 25].

Ultrasonography, as an accessible, noninvasive, and contrast-free modality, is gaining recognition for quantitative treatment monitoring in oncology. DCE ultrasonography studies have shown that early perfusion decreases at day 21 predict improved survival in patients receiving ICI [18]. Similarly, ultrafast power Doppler imaging has demonstrated the ability of ultrasonography to capture early microvascular and structural changes induced by immunotherapy [19]. The present exploratory work extends these findings by suggesting that standard B-mode images alone performed 3 weeks after starting ICI could provide prognostic information, without contrast injection or advanced acquisition. The D21 time point corresponds to the onset of immune remodeling, consistent with transcriptomic and histologic data showing B-cell activation and TLS formation within 2–3 weeks of ICI initiation [5, 6, 10].

Finally, delta-radiomics approaches, which track temporal changes in imaging metrics, could capture early therapy-induced remodeling. Here, a greater decrease in SHI between baseline and D21 was associated with poorer survival, consistent with prior CT and MRI reports linking early reductions in entropy or vascular heterogeneity to non-response [25]. These observations are hypothesis-generating and suggest (rather than demonstrate at this point) that successful immune activation may be accompanied by transient increases in local heterogeneity before later stabilization. Accordingly, our findings raise the possibility that dynamic rather than static heterogeneity measures could be more informative for early response characterization, including at the time of first radiological re-evaluation.

However, it must be noted that early imaging changes under immunotherapy could also reflect immune-cell infiltration, edema, or pseudoprogression rather than true tumor progression, which complicates the interpretation of short-term imaging biomarkers. This reinforces that our findings should be interpreted cautiously and supports the use of OS as a more robust endpoint for exploratory analyses in this setting (rather than a change in LD or PFS). More broadly, conventional size-based criteria alone may incompletely reflect treatment effects under immunotherapy, where early dimensional changes can result from biologic phenomena unrelated to true tumor burden modification. Consistent with this interpretation, no significant associations were observed between PFS and any imaging-derived metrics, including SHI values, lesion size, or their early changes. In contrast, PFS remained significantly associated with established clinical and biological factors such as histological subtype, ICI regimen, and NLR at D21 [26], supporting the internal validity of the cohort. Together, these findings suggest that short-term imaging heterogeneity changes may reflect complex immune-related dynamics that are not fully captured by early progression endpoints.

Future research should aim to validate these findings in larger, histologically homogeneous cohorts and in prospective trials integrating paired histopathology or molecular profiling. Given that early imaging changes under immunotherapy may reflect immune-cell infiltration, edema, or pseudoprogression rather than true tumor progression, careful interpretation of early heterogeneity variations is essential. If confirmed, ultrasound-derived heterogeneity assessment could provide an early, noninvasive, and low-cost indication of tumor behavior before standard CT or MRI evaluation. In particular, it may help identify patients showing early signs of disease non-control, allowing confirmation with conventional imaging (*i.e*., contrast-enhanced CT and MRI) before continuing potentially ineffective or toxic therapy. Importantly, such biomarkers should be interpreted within a multimodal clinical and radiologic framework to avoid premature discontinuation of effective treatment based solely on early imaging signals. If validated in larger cohorts, this approach could support more timely treatment adaptations and reduce unnecessary exposure to costly or burdensome interventions.

Combining B-mode heterogeneity analysis with complementary ultrasound techniques such as shear-wave elastography or Doppler-based vascular assessment could yield a more comprehensive characterization of the immune tumor microenvironment. Multimodal imaging integration, linking ultrasound-based radiomics with MRI, PET, or CT-derived metrics, or even with digital pathology (pathomics) and liquid biopsy, represents a promising avenue for noninvasive immune monitoring. Furthermore, AI-based approaches specifically designed for the joint analysis of intra- and peritumoral regions could enhance immune tumor microenvironment assessment and prediction of immune phenotypes, moving toward personalized treatment adaptation.

This study has limitations. First, this is a retrospective proof-of-concept study with a small, histologically heterogeneous population, which limits statistical power and generalizability. Although the study population was relatively homogeneous regarding major clinical factors due to inclusion in clinical trials (notably good performance status and preserved liver function), other important prognostic variables, such as tumor burden or extrahepatic disease, were not fully accounted for, and residual confounding cannot be excluded. In addition, given the limited sample size relative to the number of variables explored, our analyses may be prone to reduced statistical robustness and potential overfitting. Moreover, after adjustment for multiple comparisons, the observed associations did not remain statistically significant, further emphasizing the exploratory nature of these findings and the need for prospective validation in independent external cohorts. However, the study was based on a limited number of hypothesis-driven imaging metrics, while other variables were restricted to standard clinical descriptors or benchmarking parameters (*i.e*., NLR, LD), thereby limiting the overall dimensionality of the analysis. The wide confidence intervals observed in multivariable analyses also reflect limited precision and potential model instability related to the small sample size, warranting cautious interpretation of effect sizes. The coexistence of multiple primary tumor types with distinct biological behaviors (*i.e*., vascularity, necrosis, growth kinetics, and immune responsiveness) represents a major potential confounder, and the differences in baseline SHI values across tumor types may reflect intrinsic histologic variability rather than biological response. Second, the absence of histopathologic correlation precluded direct validation against TLS density, immune infiltration, or maturation status. Third, ultrasound measurements targeted a single lesion per patient, which may not represent the entire disease burden, particularly in cases with heterogeneous metastatic sites. Fourth, although the deep-learning-based normalization method ensured intensity standardization across acquisitions, the SHI is not yet calibrated across devices or centers, and prospective standardization will be essential for clinical translation. Fifth, manual segmentation was performed by a single reader, and no inter- or intra-observer reproducibility analysis was conducted. Prior ultrasound studies on focal liver lesions have nevertheless reported excellent reproducibility for lesion measurements, with inter-observer intra-class correlation coefficients exceeding 0.99 for volumetric assessment across multiple readers [27]. However, whether such robustness extends to derived heterogeneity metrics such as SHI remains unknown and warrants dedicated evaluation. Sixth, our cohort included multiple tumor types and different ICI regimens, which introduces biological and treatment-related heterogeneity. In the context of the limited sample size, these sources of variability cannot be disentangled or adjusted for statistically, and the results should therefore be interpreted as exploratory and hypothesis-generating rather than evidence of tumor-agnostic or treatment-agnostic effects. Seventh, although gray-level normalization accounted for subcutaneous fat and lesion size, the depth and location of liver lesions may still influence B-mode signal intensity. This residual variability could affect SHI measurements and should be addressed in future studies with standardized acquisition protocols. Furthermore, consistent with this interpretation, no significant associations were observed between PFS and any imaging-derived metrics, including SHI values, lesion size, or their early changes. In contrast, PFS remained significantly associated with established clinical and biological factors such as histological subtype, ICI regimen, and NLR at D21, supporting the internal validity of the cohort [27]. Together, these findings suggest that short-term imaging heterogeneity changes may reflect complex immune-related dynamics that are not fully captured by early progression endpoints.

In conclusion, this exploratory study provides preliminary evidence that the quantification of spatial heterogeneity from standard B-mode ultrasound at D21 on both the tumor core and its peripheral tissues offers a noninvasive, prognostic signal in patients treated with immune checkpoint inhibitors. The combined analysis of intra- and peritumoral regions appears particularly informative and may reflect the local immune architecture and its modulation under therapy. If confirmed, these preliminary results could support the concept that low-cost, accessible ultrasound imaging could contribute to precision immuno-oncology by identifying markers of treatment benefit.

## Supplementary information

**Additional File 1 :** **Table S1** Univariable overall survival (OS) and progression-free survival (PFS) analyses keeping all variables as continuous.

## Data Availability

All data generated or analyzed during this study, as well as the R script used for statistical analysis, are available from the corresponding author upon reasonable request.

## Abbreviations

CI: Confidence interval
CT: Computed tomography
D0: Day 0 (baseline)
D21: Day 21 following treatment beginning
DCE: Dynamic contrast-enhanced
HR: Hazard ratio
ICI: Immune checkpoint inhibitor
IQR: Interquartile range
LD: Longest diameter
MRI: Magnetic resonance imaging
NLR: Neutrophil-lymphocyte ratio
OS: Overall survival
PD-L1: Programmed death‑ligand 1
PET: Positron emission tomography
PFS: Progression-free survival
ROI: Region of interest
SHI: Spatial heterogeneity index
TLS: Tertiary lymphoid structures
Δ: Relative change
